# Supplementary material for: Mechanisms governing the pioneering and redistribution capabilities of the non-classical pioneer PU.1
Source: Nat Commun. 2020 Jan 21;11:402. doi: 10.1038/s41467-019-13960-2 (PMC6972792; doi:10.1038/s41467-019-13960-2)
Supplement: Supplementary file 7 — Source data [file 41467_2019_13960_MOESM7_ESM.zip › Source_Data/FigureS5/FigureS5A_MotifsCommonPeaks/homerResults.html]

/misc/data/analysis/project\_PU1/CTV1/motifs/basic/ETS1vsFLI1.DSG\_PU1.peaks.common/final - Homer de novo Motif Results


# Homer *de novo* Motif Results (/misc/data/analysis/project\_PU1/CTV1/motifs/basic/ETS1vsFLI1.DSG\_PU1.peaks.common/final)

Known Motif Enrichment Results  
Gene Ontology Enrichment Results  
If Homer is having trouble matching a motif to a known motif, try copy/pasting the matrix file into
STAMP  
More information on motif finding results: HOMER
| Description of Results
| Tips
  
Total target sequences = 8886  
Total background sequences = 38007  
\* - possible false positive  

|  |  |  |  |  |  |  |  |  |
| --- | --- | --- | --- | --- | --- | --- | --- | --- |
| Rank | Motif | P-value | log P-pvalue | % of Targets | % of Background | STD(Bg STD) | Best Match/Details | Motif File |
| 1 | C T G A C T G A T A G C T G C A C T A G A C T G C G T A C G T A T C A G G A C T T C A G | 1e-2916 | -6.715e+03 | 85.61% | 20.63% | 44.6bp (73.5bp) | Etv2(ETS)/ES-ER71-ChIP-Seq(GSE59402)/Homer(0.967)(0.968) More Information \| Similar Motifs Found | motif file (matrix) |
| 2 | A G T C C G T A A G T C A C G T A C G T A G T C A G C T | 1e-717 | -1.653e+03 | 40.46% | 12.47% | 54.7bp (74.5bp) | PU.1-IRF(ETS:IRF)/Bcell-PU.1-ChIP-Seq(GSE21512)/Homer(0.752) More Information \| Similar Motifs Found | motif file (matrix) |
| 3 | C G A T T C A G G A C T T A C G A T C G A G C T G A C T C G A T G T A C | 1e-590 | -1.360e+03 | 39.24% | 13.65% | 48.9bp (64.5bp) | RUNX1(Runt)/Jurkat-RUNX1-ChIP-Seq(GSE29180)/Homer(0.984) More Information \| Similar Motifs Found | motif file (matrix) |
| 4 | A G T C A G C T A G C T A G T C A G C T A G C T A G T C A G C T A G C T A G T C | 1e-296 | -6.827e+02 | 25.67% | 9.97% | 53.0bp (78.6bp) | Unknown4/Arabidopsis-Promoters/Homer(0.847) More Information \| Similar Motifs Found | motif file (matrix) |
| 5 | A T C G C G A T C G A T A G T C A G T C G A C T T A G C A C G T A G C T A G C T C G A T | 1e-238 | -5.498e+02 | 8.15% | 1.15% | 52.1bp (57.6bp) | SPIC/MA0687.1/Jaspar(0.798) More Information \| Similar Motifs Found | motif file (matrix) |
| 6 | C T A G A C T G A C T G C G T A C G T A G T C A A C G T C T G A G T C A | 1e-213 | -4.912e+02 | 14.65% | 4.63% | 56.5bp (67.8bp) | Pcbp2(KH)/Danio\_rerio-RNCMPT00246-PBM/HughesRNA(0.790) More Information \| Similar Motifs Found | motif file (matrix) |
| 7 | A C T G C T G A C T A G A C T G G C T A G T C A A T C G A T C G G T C A C G T A | 1e-180 | -4.162e+02 | 7.18% | 1.26% | 52.6bp (79.1bp) | RBM5(Znf)/Homo\_sapiens-RNCMPT00055-PBM/HughesRNA(0.757) More Information \| Similar Motifs Found | motif file (matrix) |
| 8 | A G T C A G C T A C G T A G T C G A T C A G C T G A C T G T A C T G A C A C T G A G T C | 1e-173 | -4.006e+02 | 10.89% | 3.15% | 48.6bp (71.2bp) | RBM5(Znf)/Homo\_sapiens-RNCMPT00055-PBM/HughesRNA(0.769) More Information \| Similar Motifs Found | motif file (matrix) |
| 9 | T C G A G C T A T A C G G C T A A C T G T C A G G T C A G C T A T A C G C G T A A C T G T C A G G T C A C T G A | 1e-165 | -3.816e+02 | 10.56% | 3.09% | 54.2bp (72.5bp) | Trl/dmmpmm(SeSiMCMC)/fly(0.726) More Information \| Similar Motifs Found | motif file (matrix) |
| 10 | T A C G T C A G A T G C T G A C C T A G A T C G G T C A C A G T T A C G A G T C A C T G T C A G | 1e-164 | -3.793e+02 | 9.51% | 2.54% | 48.3bp (63.7bp) | ETV5/MA0765.1/Jaspar(0.864) More Information \| Similar Motifs Found | motif file (matrix) |
| 11 | T G C A C T G A T G C A A T G C T A C G C T A G C G A T C G T A | 1e-153 | -3.524e+02 | 13.13% | 4.82% | 56.6bp (71.1bp) | MYB3R1(MYB)/col-MYB3R1-DAP-Seq(GSE60143)/Homer(0.763) More Information \| Similar Motifs Found | motif file (matrix) |
| 12 | C T A G T C A G G A C T A T C G G T C A T A G C C G A T T G A C C T G A A G C T | 1e-124 | -2.858e+02 | 8.86% | 2.82% | 53.4bp (61.3bp) | Atf3(bZIP)/GBM-ATF3-ChIP-Seq(GSE33912)/Homer(0.989) More Information \| Similar Motifs Found | motif file (matrix) |
| 13 | T G C A A C T G T A G C G T C A T C G A C G T A A G T C G T C A C T G A | 1e-114 | -2.640e+02 | 13.82% | 6.16% | 57.4bp (66.5bp) | FKH1/FKH1\_YPD/53-FKH1,53-FKH2(Harbison)/Yeast(0.784) More Information \| Similar Motifs Found | motif file (matrix) |
| 14 | A C T G A G T C C G A T A C G T A T C G A G T C A C T G | 1e-113 | -2.610e+02 | 11.33% | 4.54% | 56.1bp (70.8bp) | MBNL1(Znf)/Homo\_sapiens-RNCMPT00038-PBM/HughesRNA(0.900) More Information \| Similar Motifs Found | motif file (matrix) |
| 15 | G T A C G T A C C G A T A G T C A G T C A G T C G T A C A G T C C T G A A T G C | 1e-111 | -2.561e+02 | 22.09% | 12.39% | 53.5bp (63.8bp) | Egr2(Zf)/Thymocytes-Egr2-ChIP-Seq(GSE34254)/Homer(0.897) More Information \| Similar Motifs Found | motif file (matrix) |
| 16 | G A C T C T G A C G T A A G T C C G T A A C T G A G T C | 1e-97 | -2.236e+02 | 57.71% | 45.37% | 57.9bp (65.6bp) | PH0169.1\_Tgif1/Jaspar(0.766) More Information \| Similar Motifs Found | motif file (matrix) |
| 17 | A C G T A G T C C G A T A C G T C G T A A C G T A G T C C G A T | 1e-95 | -2.203e+02 | 14.87% | 7.46% | 54.3bp (67.4bp) | GATA3/MA0037.2/Jaspar(0.985) More Information \| Similar Motifs Found | motif file (matrix) |
| 18 | G T C A C A G T C T G A T C G A G T C A C T G A T C G A C T G A A C T G C T A G T C A G C T G A C T G A C T A G | 1e-94 | -2.173e+02 | 8.77% | 3.33% | 51.4bp (77.4bp) | NDD1(MacIsaac)/Yeast(0.776) More Information \| Similar Motifs Found | motif file (matrix) |
| 19 | A C G T G C A T C A G T T G C A A G T C A C T G A T C G G T A C | 1e-92 | -2.126e+02 | 10.29% | 4.36% | 51.0bp (67.8bp) | At5g08750(C3H)/col-At5g08750-DAP-Seq(GSE60143)/Homer(0.787) More Information \| Similar Motifs Found | motif file (matrix) |
| 20 | G C A T C T G A A T C G A G T C G T A C C T G A A G T C C T G A | 1e-89 | -2.050e+02 | 19.23% | 11.02% | 55.4bp (64.5bp) | MET32/MA0334.1/Jaspar(0.850) More Information \| Similar Motifs Found | motif file (matrix) |
| 21 | C G T A C G T A T A C G C T G A A C T G T C A G G T C A C G T A T A G C C G T A A C T G T G A C G C A T A C T G | 1e-86 | -1.989e+02 | 4.16% | 0.91% | 57.5bp (64.3bp) | SpiB(ETS)/OCILY3-SPIB-ChIP-Seq(GSE56857)/Homer(0.691) More Information \| Similar Motifs Found | motif file (matrix) |
| 22 | T G C A A C T G T A C G T C G A T C A G T C G A T C G A A T C G C A T G | 1e-82 | -1.910e+02 | 22.03% | 13.53% | 53.5bp (78.1bp) | SRSF1(RRM)/Homo\_sapiens-RNCMPT00163-PBM/HughesRNA(0.752) More Information \| Similar Motifs Found | motif file (matrix) |
| 23 | T A G C A T C G A T C G G C T A T C G A A C T G G T A C C G T A G C A T | 1e-77 | -1.782e+02 | 8.05% | 3.26% | 58.6bp (66.7bp) | GCR2(MacIsaac)/Yeast(0.798) More Information \| Similar Motifs Found | motif file (matrix) |
| 24 | A C G T T C G A A G T C G A T C G T C A A T G C G C T A G C A T G A T C G C T A T C G A C G T A | 1e-63 | -1.457e+02 | 2.64% | 0.49% | 46.3bp (62.6bp) | RUNX2(Runt)/PCa-RUNX2-ChIP-Seq(GSE33889)/Homer(0.733) More Information \| Similar Motifs Found | motif file (matrix) |
| 25 | G A T C C G T A G T A C G A C T A G C T A G T C G A T C G C A T T G A C G T A C G C A T A C G T | 1e-58 | -1.346e+02 | 3.63% | 1.02% | 53.6bp (70.6bp) | SpiB(ETS)/OCILY3-SPIB-ChIP-Seq(GSE56857)/Homer(0.786) More Information \| Similar Motifs Found | motif file (matrix) |
| 26 | C T A G C T A G A C T G T A C G T A C G T A C G T C A G C T A G A T C G | 1e-57 | -1.326e+02 | 23.66% | 16.22% | 56.4bp (64.6bp) | Maz(Zf)/HepG2-Maz-ChIP-Seq(GSE31477)/Homer(0.899) More Information \| Similar Motifs Found | motif file (matrix) |
| 27 | A T G C C A G T A C G T T A G C A G T C C A G T T A G C A G T C A G C T T G A C A G T C G A C T T A G C | 1e-55 | -1.270e+02 | 4.01% | 1.28% | 51.5bp (76.0bp) | PF10\_0068(RRM)/Plasmodium\_falciparum-RNCMPT00199-PBM/HughesRNA(0.704) More Information \| Similar Motifs Found | motif file (matrix) |
| 28 | C G A T G T A C C G T A C G T A C T G A A G T C A T G C A C T G G T A C | 1e-54 | -1.259e+02 | 13.01% | 7.58% | 53.9bp (69.6bp) | RUNX2/MA0511.2/Jaspar(0.839) More Information \| Similar Motifs Found | motif file (matrix) |
| 29 | G C T A C T G A A G T C T C G A C A T G G T A C G C A T C T A G | 1e-51 | -1.177e+02 | 27.14% | 19.70% | 56.3bp (65.8bp) | MyoG(bHLH)/C2C12-MyoG-ChIP-Seq(GSE36024)/Homer(0.978) More Information \| Similar Motifs Found | motif file (matrix) |
| 30 | G C A T C T G A A T C G C G A T C G A T A T G C G A T C G A C T T G A C G T C A T C G A C T G A | 1e-49 | -1.148e+02 | 4.60% | 1.73% | 56.0bp (68.7bp) | PB0162.1\_Sfpi1\_2/Jaspar(0.759) More Information \| Similar Motifs Found | motif file (matrix) |
| 31 | T G C A C T G A T C G A G T C A C G T A C T A G T A G C C G T A T C A G T G C A C T G A T C A G A G C T C G T A | 1e-43 | -9.996e+01 | 1.95% | 0.40% | 50.9bp (58.0bp) | SPI1/MA0080.4/Jaspar(0.723) More Information \| Similar Motifs Found | motif file (matrix) |
| 32 | C T A G C A T G C A T G C G T A C G T A G T A C A C G T A C T G A C G T | 1e-42 | -9.723e+01 | 6.80% | 3.40% | 56.6bp (64.5bp) | PB0165.1\_Sox11\_2/Jaspar(0.775) More Information \| Similar Motifs Found | motif file (matrix) |
| 33 | C T G A A T G C C G T A C A T G A C T G G T C A C G T A A T C G T A G C G T C A C G A T C T A G C T A G | 1e-42 | -9.722e+01 | 1.68% | 0.29% | 49.6bp (65.4bp) | Ets1-distal(ETS)/CD4+-PolII-ChIP-Seq(Barski\_et\_al.)/Homer(0.749) More Information \| Similar Motifs Found | motif file (matrix) |
| 34 | T A C G A T C G A C T G A G T C A C T G C A T G A G C T C T G A T G A C | 1e-41 | -9.583e+01 | 8.00% | 4.30% | 56.7bp (71.2bp) | hkb/dmmpmm(Papatsenko)/fly(0.866) More Information \| Similar Motifs Found | motif file (matrix) |
| 35 | A G C T T A G C A C G T A G T C A C G T A C G T G T A C T A G C A G C T A C G T | 1e-36 | -8.415e+01 | 4.13% | 1.75% | 51.9bp (79.2bp) | GCR2/MA0305.1/Jaspar(0.715) More Information \| Similar Motifs Found | motif file (matrix) |
| 36 | C T G A T A G C T G A C C T A G A T C G T C G A C T G A C T G A C T A G T C A G C T A G A T G C T A C G | 1e-36 | -8.373e+01 | 2.13% | 0.57% | 48.7bp (75.7bp) | PCBP1(KH)/Mus\_musculus-RNCMPT00239-PBM/HughesRNA(0.760) More Information \| Similar Motifs Found | motif file (matrix) |
| 37 | C G T A A C T G G T A C G T C A A C T G G T A C G T C A | 1e-35 | -8.115e+01 | 30.79% | 24.28% | 56.7bp (69.0bp) | ACE2/ACE2\_YPD/2-SWI5(Harbison)/Yeast(0.747) More Information \| Similar Motifs Found | motif file (matrix) |
| 38 | G T C A G A C T T C A G G T C A C G T A C G T A A G T C A G T C C T G A | 1e-33 | -7.760e+01 | 5.94% | 3.08% | 58.0bp (66.1bp) | IRF4(IRF)/GM12878-IRF4-ChIP-Seq(GSE32465)/Homer(0.898) More Information \| Similar Motifs Found | motif file (matrix) |
| 39 | G T C A T A G C A G C T T C G A A G T C T C G A C G T A G A C T A C G T G A T C A G T C G A T C | 1e-30 | -7.135e+01 | 1.78% | 0.47% | 56.7bp (71.1bp) | GFY(?)/Promoter/Homer(0.993) More Information \| Similar Motifs Found | motif file (matrix) |
| 40 | A C G T A T C G A G T C C A G T A T C G A G T C G A C T A C G T A G T C A G C T A T C G C G A T C T A G T A C G | 1e-30 | -6.945e+01 | 4.16% | 1.95% | 51.9bp (64.7bp) | Unknown-ESC-element(?)/mES-Nanog-ChIP-Seq(GSE11724)/Homer(0.776) More Information \| Similar Motifs Found | motif file (matrix) |
| 41 | T G C A G T C A C T G A T G A C T G C A C T A G G A T C A T G C T C G A | 1e-28 | -6.521e+01 | 11.65% | 7.84% | 59.6bp (63.3bp) | CRZ1/MA0285.1/Jaspar(0.774) More Information \| Similar Motifs Found | motif file (matrix) |
| 42 | C A G T A T G C T G C A A G T C C T A G A C G T T A C G T C G A G A T C A G C T | 1e-28 | -6.485e+01 | 6.07% | 3.39% | 57.3bp (62.5bp) | CBF1(MacIsaac)/Yeast(0.903) More Information \| Similar Motifs Found | motif file (matrix) |
| 43 | G T A C G T A C G T C A T G A C T G C A T C A G T G C A T G C A T C A G T A G C G T A C T G C A G T A C | 1e-27 | -6.382e+01 | 9.39% | 6.01% | 55.6bp (66.8bp) | CRZ1/MA0285.1/Jaspar(0.691) More Information \| Similar Motifs Found | motif file (matrix) |
| 44 | A G C T A C T G G A C T A T C G A C T G A C G T C T G A T C G A G T C A A C T G | 1e-24 | -5.580e+01 | 1.45% | 0.40% | 51.1bp (53.9bp) | RUNX2(Runt)/PCa-RUNX2-ChIP-Seq(GSE33889)/Homer(0.755) More Information \| Similar Motifs Found | motif file (matrix) |
| 45 | A G T C A C G T A C G T G A C T C G A T A G C T T G A C A C G T G A C T G T A C G T A C A C G T A C G T | 1e-22 | -5.227e+01 | 1.65% | 0.54% | 58.2bp (72.3bp) | Unknown4/Arabidopsis-Promoters/Homer(0.629) More Information \| Similar Motifs Found | motif file (matrix) |
| 46 | C G T A A C T G A G T C C G T A A C T G C G T A A C G T | 1e-22 | -5.212e+01 | 11.34% | 7.96% | 58.6bp (63.7bp) | Tal1(0.788) More Information \| Similar Motifs Found | motif file (matrix) |
| 47 | T A G C C G A T T A C G T G C A C G A T T G C A C T G A A T G C T G A C A C T G G T A C C G T A | 1e-21 | -4.878e+01 | 0.82% | 0.14% | 49.6bp (50.1bp) | Bgb::run/MA0242.1/Jaspar(0.780) More Information \| Similar Motifs Found | motif file (matrix) |
| 48 | A C T G A C T G A G T C T C G A A C T G A C G T A C G T | 1e-21 | -4.874e+01 | 6.43% | 3.99% | 54.7bp (71.7bp) | MYB(HTH)/ERMYB-Myb-ChIPSeq(GSE22095)/Homer(0.930) More Information \| Similar Motifs Found | motif file (matrix) |
| 49 | T A G C C G T A A C G T A C T G A G T C G T C A A C G T G A C T A G T C A C G T A T C G G A C T T C A G A T G C | 1e-21 | -4.869e+01 | 0.75% | 0.12% | 52.9bp (49.5bp) | YAP5(MacIsaac)/Yeast(0.644) More Information \| Similar Motifs Found | motif file (matrix) |
| 50 | T A G C G A T C C T G A T A G C A G C T G T C A C T A G C T A G A C T G A C T G A T C G G A T C C T A G | 1e-20 | -4.714e+01 | 1.61% | 0.55% | 58.0bp (67.8bp) | BORIS(Zf)/K562-CTCFL-ChIP-Seq(GSE32465)/Homer(0.929) More Information \| Similar Motifs Found | motif file (matrix) |
| 51 | A C G T A C T G A C G T A C G T A T G C A G T C A C G T A G T C G A C T T A G C G T A C A T C G | 1e-18 | -4.217e+01 | 1.32% | 0.43% | 57.4bp (72.3bp) | SPIB/MA0081.1/Jaspar(0.800) More Information \| Similar Motifs Found | motif file (matrix) |
| 52 | A C G T A C T G G T C A C G A T T C G A T G C A G T A C C G T A C G A T A G T C C G A T | 1e-17 | -4.097e+01 | 0.70% | 0.12% | 46.8bp (57.9bp) | PB0125.1\_Gata3\_2/Jaspar(0.829) More Information \| Similar Motifs Found | motif file (matrix) |
| 53 | G A T C C G A T A T C G T G C A A G C T C T G A C T G A C T A G T C A G G T C A C T G A T C A G | 1e-15 | -3.681e+01 | 0.92% | 0.25% | 61.2bp (73.5bp) | PB0022.1\_Gata5\_1/Jaspar(0.800) More Information \| Similar Motifs Found | motif file (matrix) |
| 54 | G A C T A G C T G A T C A G C T A G T C C T G A G A T C T C A G T C G A A C T G G T C A C G A T A G T C | 1e-15 | -3.601e+01 | 1.01% | 0.30% | 64.2bp (63.0bp) | ZBTB33(Zf)/GM12878-ZBTB33-ChIP-Seq(GSE32465)/Homer(0.893) More Information \| Similar Motifs Found | motif file (matrix) |
| 55 | A C G T A T G C A C G T A G T C G A C T G A C T G A T C G A T C G A C T A T G C A G T C C G A T A C T G | 1e-13 | -3.035e+01 | 1.05% | 0.37% | 57.0bp (71.9bp) | SeqBias: GA-repeat(0.712) More Information \| Similar Motifs Found | motif file (matrix) |
